# Mesenchymal Stem Cells in Tissue Growth and Repair 

**Published:** 2011

**Authors:** N.I. Kalinina, V.Yu. Sysoeva, K.A. Rubina, Ye.V. Parfenova, V.A. Tkachuk

**Affiliations:** Department of Fundamental Medicine, Lomonosov Moscow State University

**Keywords:** mesenchymal stem cells, tissue regeneration, differentiation, cell therapy

## Abstract

It has been established in the recent several decades that stem cells play a crucial role in tissue renewal and regeneration. Mesenchymal stem cells (MSCs) are part of the most important population of adult stem cells. These cells have hereby been identified for the very first time and subsequently isolated from bone marrow stroma. Bone marrow-derived MSCs have been believed to play the role of a source of cells for the renewal and repair of connective tissues, including bone, cartilage and adipose tissues. Cells similar to bone marrow-derived MSCs have now been identified in all postnatal tissues. Data on the distribution and function of MSCs*in vivo*collected using novel approaches pertaining to the identification of MSCs*in situ*, to their isolation from tissues, and finally to the determination of their biological properties have enabled successful revision of the role of MSCs in various organs and tissues. This review summarizes our own, as well as others’, data concerning the role of MSCs in the regulation processes of tissue repair and regeneration. In our opinion, MSCs provide the connection between the blood-vascular, immune, endocrine, and nervous systems and tissue-specific stem cells in the body.

## INTRODUCTION

The conception of tissue renewal being facilitated by a self-sustaining pool of stem cells was first formulated in the study of hematosis over a century ago [1]. However, the existence of hematopoietic stem cells (HSCs) from which all blood cell types stem was confirmed experimentally only in the middle of the 20th century [2]. Until then, it was believed that the cellular composition of postnatal tissues was replenished as a result of the division of specific (differentiated) cells. The concept that the renewal occurrs as a result of the activity of stem cells was considered a mechanism limited only to blood cells; a unique, rapidly renewing tissue containing a great number of functionally heterogeneous cell types. It has now been proven that the maintenance and replenishment of the cellular composition in almost all tissues of the human body (including skin and intestinal epithelium, liver, skeletal muscles, and myocardium) occur through the proliferation and differentiation of the corresponding tissue-specific stem cells (TSCs). However, along with the tissue-specific stem cells, other stem cells, known as mesenchymal stem cells (MSCs), have been identified in mammalian tissues.

**Fig. 1:**
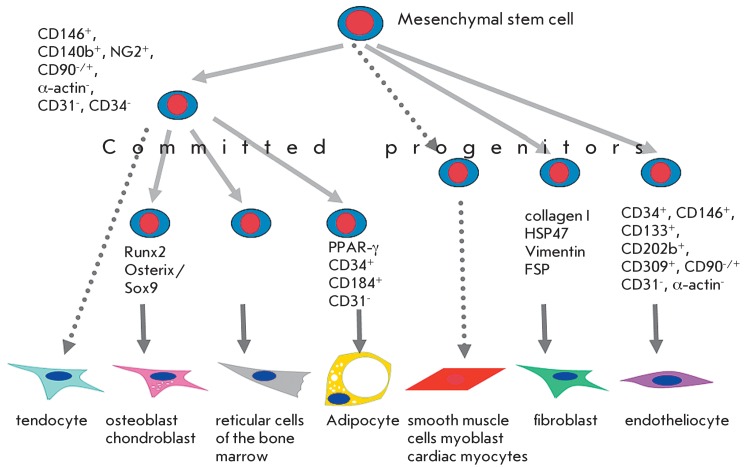
Model of mesenchymal hierarchy. The hypothetic scheme, according to which MSCs undergoes several discrete stages of differentiation, thereby producing various types of connective tissue cells for the entire life.

The first body of data indicating the presence of postnatal stem cells, along with hematopoietic stem cells, in bone marrow was obtained by means of several approaches. The first indication was the occurrence of osteogenesis in experimental models following the intraperitoneal transplantation of cultivated bone marrow stromal cells [3]. The second indication was the isolation of a cell population from the bone marrow that differs from hematopoietic stem cells but exhibits properties typical of stem cells. During cultivation, these cells cloned (the colonies of fibroblast) [4], maintaining their ability to differentiate into a variety of cell types (osteoblasts, adipocytes, and chondrocyte) [5].

The assumption was, by analogy with HSCs, that bone marrow-derived MSCs were at the top of the mesenchymal hierarchy [6, 7] ( *Fig. 1* ). It was suggested that, throughout life, descendants of these cells undergo several discrete stages of differentiation, thereby spawning the various cells found in connective tissues, i. e.: in bone and adipose tissues, tendons, cartilages, and smooth muscles [8]. Later, the cells with phenotypic characteristics and differentiation potential similar to bone marrow-derived MSCs were isolated from almost all the embryonal and postnatal tissues of mammals, birds, and amphibians [9, 10]. On the basis of these observations, a theory emerged, postulating that bone marrow is a deposit of both hematopoietic and mesenchymal stem cells. However, the suggestion that the renewal of connective tissues over the whole body depends on the activity of bone marrow-derived MSCs as yet remains unconfirmed [11]. 

## CULTIVATED MULTIPOTENT MESENCHYMAL STROMAL CELLS

Identifying MSCs and analyzing them directly in tissues are very complicated tasks. Hence, most conclusions relating to the biological properties of MSCs are made on the basis of a study of stromal cell populations isolated from various tissues; these stromal cells possess the ability to attach culture plastic and to differentiate in osteogenic, adipogenic, and chondrogenic directions *in vitro* [12]. Despite the fact that, by their ability to renew and differentiate in various directions, these cells form a rather heterogenic population [13], they are also referred to as MSCs. The International Society for Cellular Therapy (ISCT, Vancouver, Canada) has suggested using the term “multipotent mesenchymal stromal cell” (MMSC) in order to separate such cultivated cells from MSCs *in situ* . According to the minimal criteria developed by the ISCT, MMSCs should have the ability to attach to plastic during cultivation in ambient conditions, to express CD105, CD73, and CD90 marker antigens on the surface, but they should not contain CD45, CD34, CD14 or CD11b, CD79α or CD19, and HLA-DR, and they also should differentiate into osteoblasts, adipocytes, and chondroblasts *in vitro* [14]. At present, bone marrow and adipose tissues are considered to be the most promising sources of MMSCs for use in the study of the biological properties of these cells and for their application in regenerative medicine. However, multipotent mesenchymal stromal cells have also been isolated from other tissues, including skin, thymus, spleen, and endometrium [15]. It should be taken into account that MMSCs isolated from different postnatal and embryonal tissues differ from each other in the following ways: by their ability to form colonies, by gene expression, and by their differentiation potential even if they had been cultivated under the same conditions [9, 15–18]. From these distinctions, questions as to the extent to which the cells (selected based on their ability to attach to plastic and grow in ambient conditions of cultivation) are biologically equivalent to one another and whether or not these differences are the result of various biological functions of MSCs in the corresponding tissues arise [12]. 

## MULTIPOTENCY OF MMSCs

In addition to their ability to differentiate into osteoblasts, chondroblasts, and adipocytes *in vitro* [7], MMSCs give rise to bone or cartilage after ectopic transplantation *in vivo* in animal models [3], as well as mediate the regeneration of bone tissue following injuries [11] and when genetic defects occur in osteogenesis (osteogenesis imperfecta) [19]. Moreover, many investigations have shown that MMSCs have the ability to differentiate into a variety of cells having mesodermal, ectodermal, and endodermal origins, including endothelial cells [20], cardiac myocytes [21], hepatocytes [22], and neural cells [23]. A number of authors, however, conceding the ability of MMSCs to differentiate into osteoblasts, chondroblasts and adipocytes, continue to doubt the ability of MMSCs to differentiate into cells of other germ layers (endodermal and ectodermal) both *in vitro* and *in vivo* [12]. It has thus been shown that, post transplantation, bone marrow-derived MMSCs can integrate into the tissues of a recipient by fusion with resident cells [24] rather than through differentiation into the cells typical of the particular tissue. 

The differences in the assessment of the differentiation potential of cultivated MMSCs can be explained by the dissimilar quantitative and qualitative compositions of progenitor cells in tissues from which they were obtained. Firstly, the isolated population of MSCs is heterogenic and includes cells with differing morphologies and cells exhibiting different proliferative and differentiation abilities *in vitro* and *in vivo* [25, 26]. Furthermore, the multipotency of MMSCs could disappear as they are being cultivated [25]. Thus, the clones of umbilical-cord-derived MMSCs, which differ from each other by their degree of self-renewal and by their differentiation potential *in vitro* , produce daughter clones that gradually lose their multipotency [25]. 

Secondly, it is possible that the newly isolated populations of MMSCs can contain progenitor cells already committed in various differentiation directions. Thus, according to the results of a cytometric analysis, adipose tissue contains at least five progenitor cells differing in the expression of marker antigens: sub-endothelial progenitor cells (CD146 ^+^ , CD140b ^+^ , NG2 ^+^ , α-actin ^-^ , CD31 ^-^ , CD90 ^-/+^ , and CD34 ^-^ ), supra-adventitional progenitor cells (NG2 ^+^ , CD90 ^+^ , CD34 ^+^ , CD146 ^-^ , CD31 ^-^ , and α-actin ^-^ ), and transient progenitor cells (CD146 ^+^ , CD34 ^+^ , NG2 ^+^ , CD90 ^+^ , and CD31 ^-^ ), preadipocyte progenitor (CD34 ^+^ , CD184 ^+^ , and CD31 ^-^ ), and endothelial progenitor cells (CD34 ^+^ , CD146 ^+^ , CD133 ^+^ , CD202b ^+^ , CD309 ^+^ , CD31 ^-^ , CD90 ^-/+^ , and α-actin ^-^ ) [26]. In this author’s opinion, the fact outlined above explains the frequent presence of endothelial islands (which disappear with cultivation) in a primary culture of MMSCs and explains the spherical shape of the colonies that are formed by small, and rapidly dividing rounded cells and large, very slowly dividing spread cells [27]. 

**Fig. 2:**
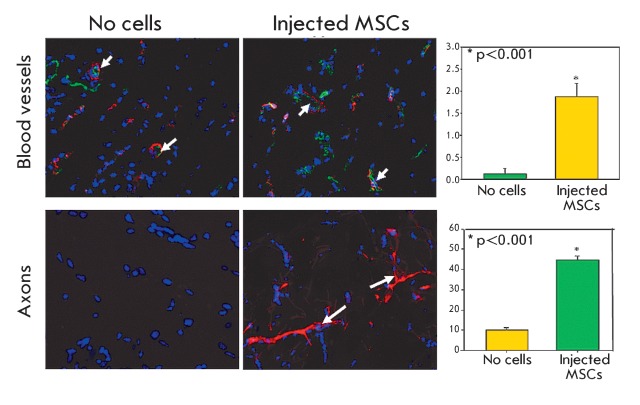
Stimulation of growth of blood vessels and axons under the action of MSCs. Adipose tissue-derived MSCs were transplanted as subcutaneous matrigel implants in syngeneic mice. Blood vessel (upper panels) and axon (lower panels) densities were evaluated by immunofluorescent staining of frozen sections with antibodies against the markers of endothelium (CD31, green fluorescence) and pericytes (NG2, red fluorescence), or axonal cytoskeleton protein (NF200, red fluorescence). Cell nuclei are counterstained by DAPI. Short arrows indicate mature blood vessels, long arrows indicate axons. The diagrams represent the results of morphometry for the total length of blood vessels (upper diagram) and axons (lower diagram) [33, 34].

Thirdly, along with MSCs, all tissues contain minor subpopulations of pluripotent cells that have a wider spectrum of differentiation abilities, such as minute embryonic-like (VSEL) cells [28], multipotent adult progenitor cells (MAPCs) [29], and multilineage differentiating stress-enduring (MUSE) cells [30]. Our data indicate the presence of very small embryonic-like cells in a newly isolated population of MMSCs; but the contribution of these minor populations to the differentiation potential of the MMSC culture remains unclear, since it is unknown whether they retain their growth and differentiation ability under cultivation conditions.

The disagreement in the analysis of the multipotency of MMSCs can also be attributed to the use of different techniques for isolation and cultivation, as well as the various assessment criteria of differentiation.

## MMSCs AND TISSUE GROWTH

Transplantation of MMSCs stimulates the regeneration of tissues, including bone, skeletal muscles, myocardium, skin, liver, and peripheral nerves. According to our data, this occurs owing to both the integration of transplanted MMSCs into the recipient’s tissues and the secretory activity of these cells [31]. It was demonstrated that transplanted MSCs integrate the endothelial lining of growing capillaries and the periendothelial space of newly formed blood vessels, thereby stabilizing them.

MSCs are an important source of growth factors and cytokines, which participate in the regulation of tissue regeneration. Thus, MSCs produce factors in the bone marrow that are necessary for the self-sustenance of hematopoietic stem cells and keep them in a niche; we can refer to such factors as SDF-1α (the stromal factor-1α), SCF (stem cell factor), angiopoietin -1, and interleukin-7 [32]. It was established in our laboratory that MSCs produce angiogenic and neurotrophic growth factors, including VEGF (vascular endothelial growth factor), bFGF (basic fibroblast growth factor), HGF (hepatocyte growth factor), angiopoietin, NGF (nerve growth factor), BDNF (brain-derived neurotrophic factor), and GDNF (glial cell line-derived neurotrophic factor) [33, 34]. The angiogenic growth factors produced by MMSCs in the transplantation region stimulate the division of endothelial cells, their migration, and the formation of blood vessels. In addition, the factors produced by MMSCs promote the mobilization of endothelial progenitors from the bone marrow, which participate in the formation of new blood vessels [34, 35]. Simultaneously, the neurotrophic factors produced by MMSCs stimulate both the growth and renewal of nerve endings [33]. Thus, MSCs can mediate the coordinated regulation of the growth of blood vessels and nerves during regeneration and remodeling of tissues ( *Fig. 2* ). 

According to our data, MMSCs produce the following factors necessary for the functional maturation of blood vessels and their stabilization: bFGF, PDGF-BB (platelet-derived growth factor BB), and TGF-β (transforming growth factor beta) [18, 34, 36]. PDGF-BB initiates branching to them of growing blood vessels and migration of pericytes, smooth muscular cells, and mesenchymal cells; while TGF-β stimulates the differentiation of the smooth muscular cells and the production of the extracellular matrix components of the vascular wall [37].

The amount of growth factors and cytokines produced in MSCs significantly increases when organs and tissues are damaged. Hypoxia causes coordinated changes in the expression of genes in MMSCs: the level of mRNA for the proangiogenic factors, such as VEGF, PIGF, HGF, bFGF, PDGF-BB, and TGF-β, increases by a factor of 2–4; and the mRNA level for anti-angiogenic factors, such as PAI-a, angiostatin, and thrombospondin, decreases more than twofold [34]. Furthermore, MMSCs secrete neurotrophic factors, including NGF, BDNF, and GDNF, which are responsible for the stimulation of growth and regeneration of the nerve filaments that are a result of the transplantation of MMSCs [33, 38].

The secretory activity of MMSCs also affects their immunomodulating properties. In several animal models, it was revealed that the injection of cultivated MMSCs causes immunosuppression *in vivo* [39]. The immunosuppressive effect of MMSCs is based on the suppression of the function of immune cells by means of the following: through the activation of T cells, via the differentiation of dendrite cells, through the proliferation of B cells, and by means of the cytolytic activity of natural killers. The immunosuppressive properties of these cells are mediated by the secretion of soluble factors, including interleukin-10, prostaglandin-E2, nitric oxide, TGF-β _1_ , galectin-1 and galectin-3, as well as by the indirect intercellular contact of MMSCs with immune cells [40]. Moreover, MMSCs can promote the differentiation of naive T helpers into regulatory T cells and the migration of mature regulatory T cells [39, 40]. The colocalization of bone marrow-derived MSCs with dendrite cells and circulatory B cells in the perisinusoid space [41, 42] enables the reasonable assumption that MSCs are involved in the regulation of the functional activity and maturation of immune cells. 

## MMSCs AND TUMOR GROWTH

Tumor growth and angiogenesis are examples of pathologic remodeling of tissues. Mesenchymal cells are known to interact with tumor cells, both sustaining and inhibiting tumor growth *in vitro* and *in vivo* . It has thus been revealed that, *in vitro* , bone marrow-derived MMSCs stimulate the proliferation of pancreatic cancer cells [43], while MMSCs from the subcutaneous adipose tissue suppress the proliferation of primary leukemia cells [44]. In animal models, it was found that MMSCs stimulate the retention and growth of tumor cells when they are injected in conjunction with melanoma cells [45–47], breast cancer [48], prostate cancer [49], as well as bowel cancer [50, 51]. In addition, they also increase the probability of metastasis formation [52]. In all likelihood, the stimulating effect of MMSCs occurs owing to the secretion of chemokines (CCL5, SDF-1α) and agiogenic growth factors (VEGF) and to their anti-apoptotic influence on tumor cells. In addition, MMSCs have the ability to migrate to tumor tissue and participate in the formation of its stroma. Thus, in tumors, MMSCs differentiate into fibroblasts, endothelial cells, and pericytes, thereby stimulating tumor growth [46, 53]. MMSCs are also capable of secreting cytokines, growth factors, and angiogenic factors [34, 43]; this stimulates tumor growth through increasing their vascularization. 

It should be noted, however, that in these and other models, MMSCs can actually suppress tumor growth under particular conditions. There are several underlying processes for this MMSCs effect, such as the stimulation of the inflammatory reaction in the recipient’s body in the case of colorectal cancer [50]; the activation of the Akt and Wnt signaling pathways in Kaposi’s sarcoma cells, hepatoma cells, and breast cancer cells [54–56]; cell cycle arrest in G1-phase occurring in pancreatic cancer cells, hepatoma cells, and lymphoma cells [44, 57]; induction of apoptosis in tumor and endothelial cells in hepatoma and non-Hodgkin’s lymphoma [44, 58]; and the suppression of angiogenesis in melanoma of B16F10 mice [57]. The systematic injection of MMSCs to experimental animals suffering from induced mature mancreatic cancer led to the apoptosis of tumor cells *in vitro* and to the suppression of tumor growth *in vivo* [57]. 

The data obtained during the study of the effect of MSCs on tumor growth indicate that the activation of MSCs, their directed migration, and their differentiation into the cells of connective tissue and blood vessels, as well as their interaction with immune cells, are important processes in oncogenesis.

## IDENTIFICATION OF MSCs *in vivo*

The distribution of MSCs in tissues has remained unclear for a considerable period of time [13, 32], since the fraction of such cells in tissues is rather small, and the unique immunophenotype distinguishing them from other cells had not been determined. New marker antigens of MSCs were determined via the assessment of the ability of cells, which were isolated from tissues in accordance with their ability to express particular proteins, for self-renewal and differentiation *in vivo* . 

It was recently revealed that in the bone marrow of mice, the following cells satisfy the criteria for MSCs: cells expressing Sca-1 (stem cell antigen-1) and PDGFRα (platelet-derived growth factor receptor α). These cells formed bone tissue and the functional stroma for HSCs during their heterotopic transplantation under the skin and osteoblasts, reticular cells, and adipocytes in the bone marrow after their systematic transplantation to an exposed recipient [13]. It was established using the same approach, that nestin can also be a marker of bone marrow-derived MSCs [32]; this conclusion was drawn since the cells expressing nestin possessed the ability to self-renew *in vitro* and *in vivo* , differentiated into osteoblasts and chondrocytes in the bone marrow *in vivo* , and formed a hematopoietic microenvironment when transplanted under the skin [59]. 

**Fig. 3:**
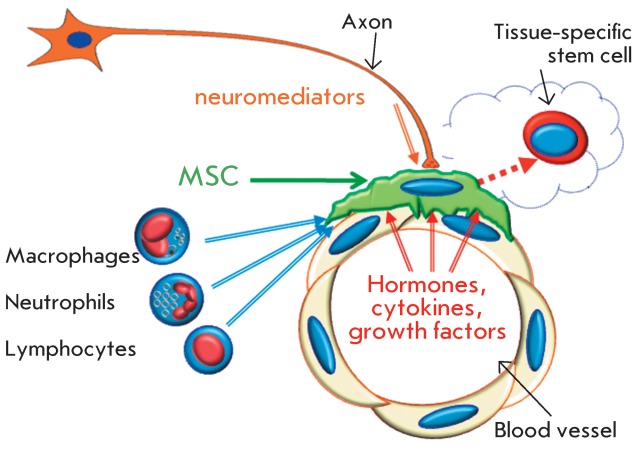
Hypothetic scheme showing the interaction of MSCs with axons, endothelial cells, leukocytes, and tissue-specific stem cells (see details in the text).

By using new marker antigens (CD146, Sca-1, and PDGFRα), it was shown that, *in situ* , MSCs accumulate in the immediate vicinity of blood vessels; in particular in the adventitia of the arteries supplying blood to the bone marrow. Moreover, the cells, which were isolated in accordance with their perivascular localization and the ability to express pericyte markers (NG2 [chondroitin sulfate proteoglycan], CD146, and PDGFRβ) from various human organs and tissues, including the fetal and postnatal skin, the pancreas, the heart, the lungs, the bone marrow, and placenta, had the ability to self-maintain and also possessed differentiation potential and expression profiles that are typical of MSCs [60]. 

 It is now widely believed that the cells producing the marker antigens of MSCs and pericytes *in vivo* are found in close proximity to blood vessels, in adipose tissue, and in dental pulp [61, 62]. 

Despite the similarities in the localization and ability to express surface markers, the ability to differentiate into osteoblasts, chondrocytes, adipocytes, myocytes, and smooth muscle cells *in vitro* and form the foci of ectopic osteogenesis *in vivo* [60], the problem of the biological equivalence of MSCs and pericytes remains unresolved. 

It remains thus unclear whether MSCs incorporate all of the functions of pericytes *in vivo* : e. g., the stabilization of capillaries, phagocytosis, and the regulation of the permeability and tonus of blood vessels, including those functions that are controlled through the reception of signals from the sympathetic nervous system [63]. The periendothelial space of blood vessels in all likelihood contains heterogenic cell populations. Apparently, only a portion of pericytes can belong to MSCs *in vivo* . The perivascular localization is a typical feature of not only MSCs; it has been noted in resident stem cells (or progenitor cells), HSCs, preadipocytes, and stem cells in the skeletal muscles of the bone marrow, in adipose tissues, and skeletal muscles [64–67]. The presence of MSCs in the periendothelial space indicates that endothelium is an important component of the MSC niche, which can regulate their functional activity.

## MSCs ARE COMPONENTS OF THE TISSUE-SPECIFIC CELL NICHE

Studying the functions of MSCs in various tissues has become a topical research task. In the bone marrow, MSCs provide not only the renewal of stroma, but they are also an important regulator of hematosis and the functions of HSCs. HSCs in the bone marrow have a particular microenvironment (niches), which is comprised of non-hematopoietic cells, soluble factors and proteins of the territorial matrix, which regulate the process of hemopoiesis [69]. At the beginning of studies of the role of MSCs, these cells *in vivo* were found to be a source of osteoblasts, adipocytes, and reticular cells; altogether, these cells make up the niche for HSCs, which participate in hemopoiesis [3]. In addition, MSCs are the first to populate the sites of fetal hemopoiesis, transmitting the regulatory signals causing the migration of HSCs to those sites [70]. Cultivated bone marrow-derived MMSCs are also capable of maintaining the survival and proliferation of HSCs *ex vivo* [70]. 

Osteoblasts are necessary components of the hematopoietic microenvironment [71–72]. The number of osteoblasts in the bone marrow positively correlates with the number of HSCs [73]; i. e., around 14% of HSCs are located in the immediate vicinity of the osteoblast-lined endosteum [65]. The ability of osteoblasts to regulate the self-maintenance and activation of HSCs has not yet been confirmed unequivocally. However, data showing that these cells regulate the differentiation of HSCs into granulocytes and B lymphocytes through the secretion of soluble growth factors and cytokines, such as LIF-1 (leukemia inhibitory factor-1), GM-CSF (granulocyte macrophage colony-stimulating factor), SDF-1, and interleukin-6 has been obtained *in vitro* [69]. Along with osteoblasts, the stroma of the bone marrow contains adipocytes, which also originate from MSCs. It is of interest that adipocytes function in the bone marrow as negative regulators of hemopoiesis, acting through a mechanism that is still unknown [74]. Consequently, bone-marrow MSCs play the role of a source for two types of cells (osteoblasts and adipocytes), which exhibit antagonist activity in the regulation of HSCs. It remains unclear what is the deciding factor concerning the direction in which MSCs differentiate, and how the balance between the production of osteoblasts and adipocytes would affect hemopoiesis. [12]. 

MSCs can also regulate the hemopoietic microenvironment by arranging a vasculature in the bone marrow; the latter is a necessary structural and functional component of HSC niches [69]. The existence of dual stem-cell niches containing two types of stem cells, HSCs and MSCs, which directly interact in the perivascular spaces of the brain marrow, was confirmed in two independent studies. In particular, it was established that the majority of HSCs are located at a distance of below 30 µm (~5 diameters of a HSC) from the reticular cells which produce a large amount of SDF-1α and nestin [13, 32]. As was mentioned above, these cells are MSCs or their closest descendants, since they are capable of both self-maintenance and differentiation in the osteogenic and adipogenic directions both *in vitro* and *in vivo.*

Nestin-positive MSCs express factors are required to keep HSCs in their niche and for them to self-maintain; accordingly, their number should be 50–700 times higher than that of the other stromal cells of the bone marrow. In addition, nestin-positive MSCs maintain the growth of HSC colonies *in vitro* , and their removal causes a drastic fall in the amount of HSCs *in vivo* . 

The distinctive features of nestin-expressing MSCs from the bone marrow are the expression of β3-adrenoreceptors and their ability to respond to signals from the nervous system. The agonists of β3- and β2-adrenoreceptors induce suppression in the expression of SDF-1α, SCF, angiopoetin-1, and interleukin-7 by nestin-exressing MSCs; the latter, in turn, leads to the mobilization of HSCs. Thus, hemopoiesis is regulated by the nervous system in this manner.

It has been well-known since the time of Virchow that pericytes with periendothelial localization can be targets for axon endings, thereby playing a key role in the transmission of signals from the nervous system to the vascular system [75]. As was noted above, part of pericytes belong to MSCs. Therefore, the MSC function of connecting the compartment of HSCs and the nervous system is in good agreement with their perivascular localization ( *Fig. 3* ). 

Bone-marrow-derived MSCs are also targets for innate immunity cells, such as macrophages. In contrast to the nervous system stimulating mobilization, bone-marrow-derived macrophages promote the retention of HSCs in the niche [76].

It remains unclear whether MSCs from other tissues are part of the niches for tissue-specific resident stem cells, and if they mediate the action of the nervous system in these niches. Thus, MSCs isolated from myocardium were found to be capable of stimulating the survival and proliferation of cardiac stem cells *in vitro* [77]; however, the questions of whether and how these cells interact with tissue-specific stem cells *in vivo* remains unanswered [78]. In both the small intestine and skin, the populations of MSCs responsible for the renewal of these tissues after injury were identified. However, the degree of importance of their interaction with tissue-specific stem cells for the processes of tissue renewal has yet to be studied. It is at least known, however, that bone-marrow MSCs are a necessary component of the perivascular niche for the tissue-specific resident stem cells (in this case HSCs) which enable integration between signals from the nervous and immune systems and the peripheral blood flow. 

## CONCLUSIONS

The results presented in this review allow us to suggest that there are two types of MSCs in the body: bone-marrow-derived MSCs circulating in the blood, which participate in tissue repair upon injury, and resident MSCs, which are located in the perivascular region of all organs and tissues of the body and regulate physiological tissue renewal and the maintenance of tissue homeostasis. MSCs are important participants in the processes of tissue renewal and regeneration. Firstly, they regulate the self-maintenance and differentiation of tissue-specific stem cells. Secondly, MSCs stimulate growth, as well as stabilize blood vessels and nerves in the processes of tissue repair. Thirdly, the interaction between MSCs and lymphocytes, endothelial cells and axons facilitates the integration of the neurohumoral signals which regulate tissue renewal and repair. 

## Abbreviations

HSC: hematopoietic stem cell
MSC: mesenchymal stem cell
MMSC: multipotent mesenchymal stromal cell
TSC: tissue-specific stem cell
